# Supplementary material for: Enhanced inhibition of MHC-I expression by SARS-CoV-2 Omicron subvariants
Source: Proc Natl Acad Sci U S A. 2023 Apr 10;120(16):e2221652120. doi: 10.1073/pnas.2221652120 (PMC10120007; doi:10.1073/pnas.2221652120)
Supplement: Supplementary file 1 — Appendix 01 (PDF) [file pnas.2221652120.sapp.pdf]

PNAS

Supporting Information for
Enhanced inhibition of MHC-I expression by SARS-CoV-2 Omicron subvariants

Miyu Moriyama[1], Carolina Lucas[1], Valter Silva Monteiro[1], Yale SARS-CoV-2 Genomic Surveillance Initiative[a], and Akiko Iwasaki[1,2,3]

Corresponding Author: Akiko Iwasaki

Email: akiko.iwasaki@yale.edu

This PDF file includes:

Figures S1 to S4
Complete list of the Yale SARS-CoV-2 Genomic Surveillance Initiative authors

A

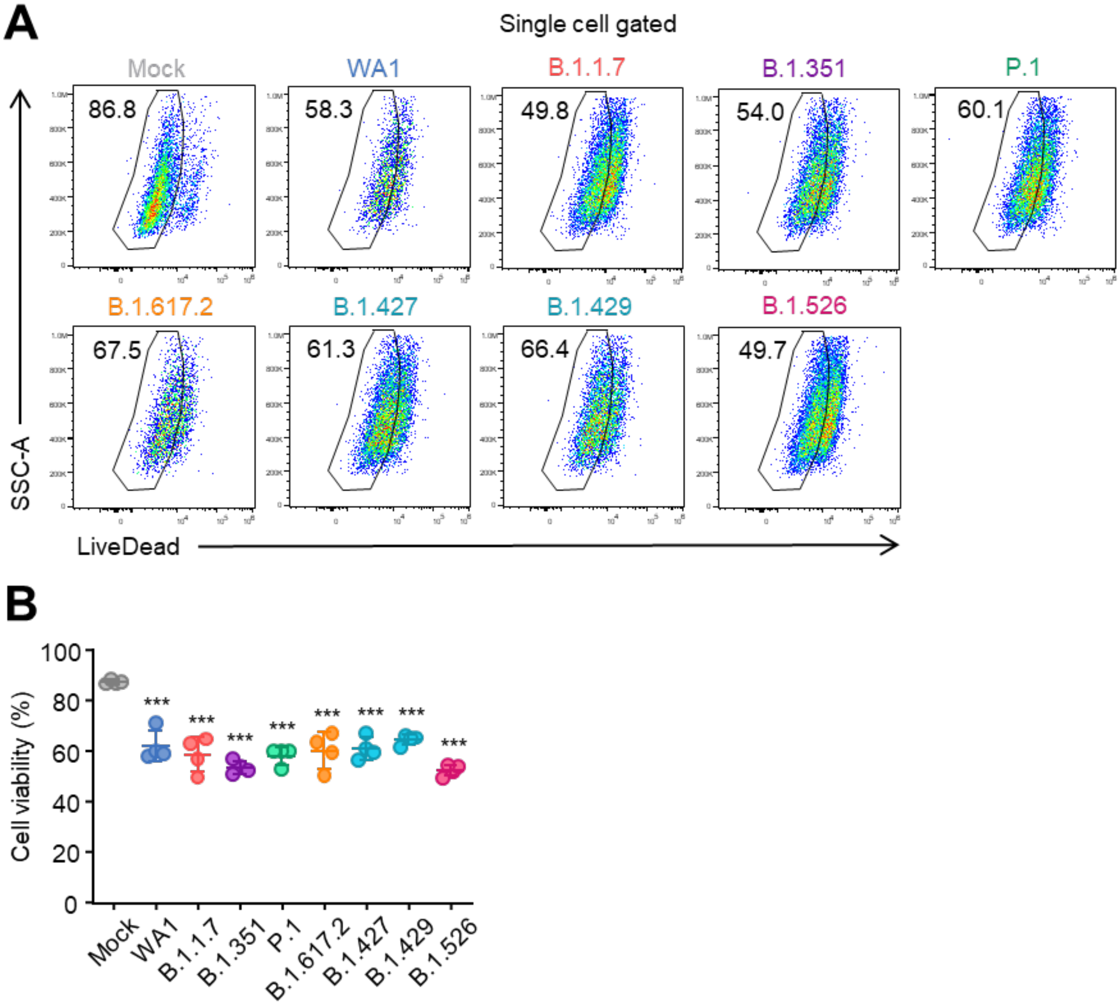

Fig. S1. Cell viability during infection with SARS-CoV-2 variants.
(A-B) Calu-3 cells were infected with SARS-CoV-2 variants at MOI 0.3 for 40h, and the cell viability was analyzed by FACS. (A) Representative FACS plot and (B) statistics are shown. Data are mean ± s.d. Data are representative of three independent experiments. ***, $p < 0.001$

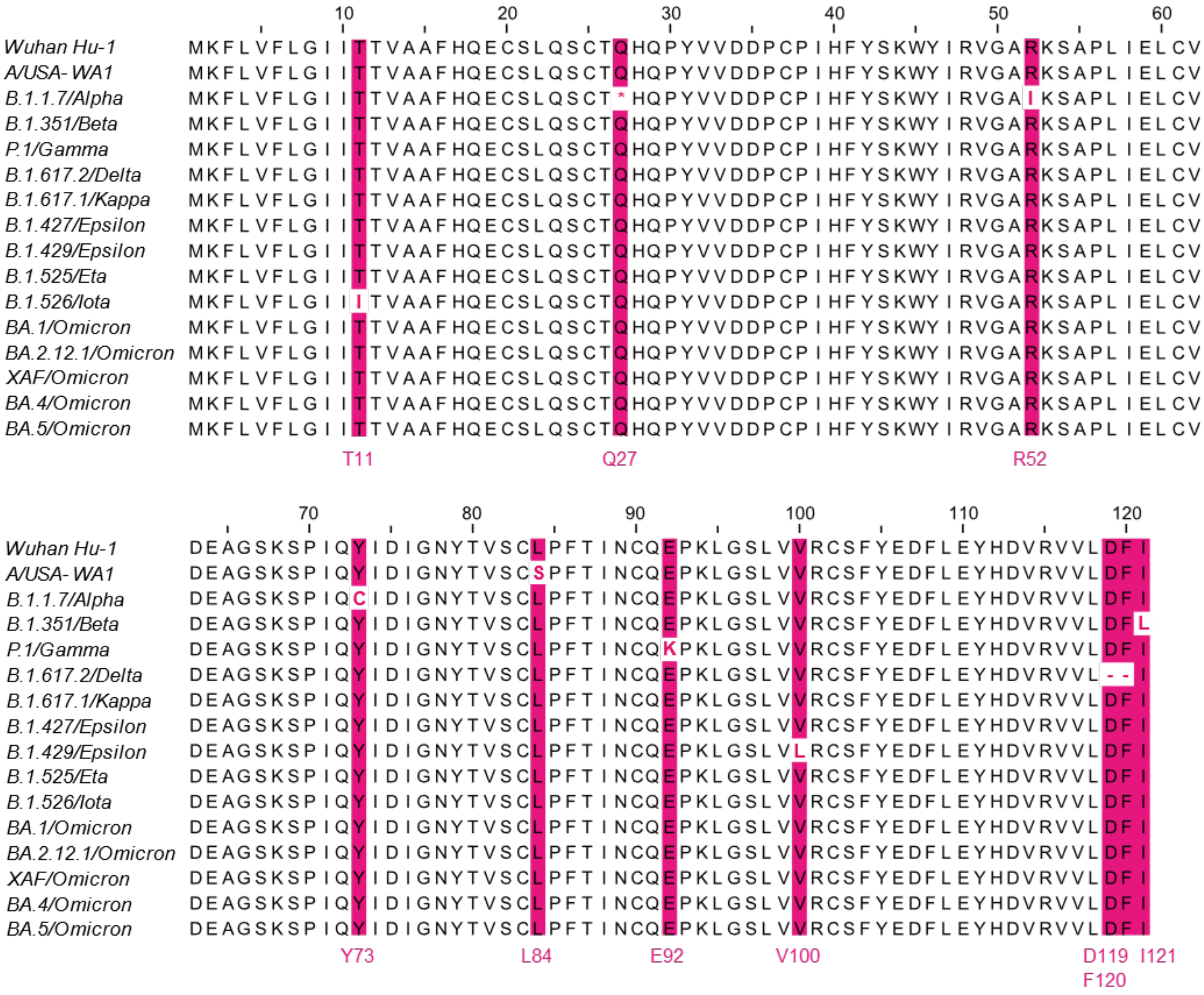

Fig. S2. Comprehensive screening of mutations in ORF8 in variants of concern/interest

Multiple sequence alignment of ORF8 protein from SARS-CoV-2 variants. Amino acid residues where mutations are noted are colored in magenta.

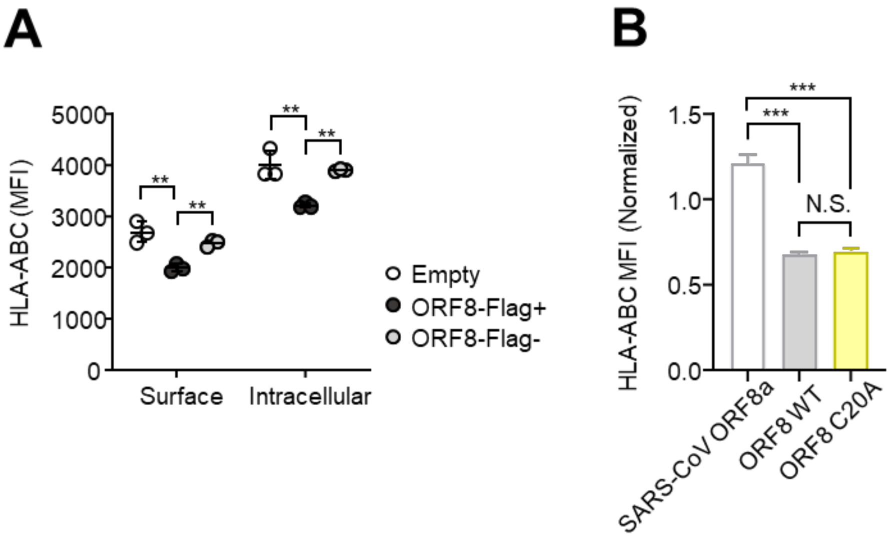

Fig. S3. MHC-I evasion by SARS-CoV-2 ORF8

(A) HEK293T cells were transfected with plasmid encoding C-terminally Flag-tagged SARS-CoV-2 ORF8 WT or empty vector. Forty-eight hours after transfection, cells were collected and analyzed for the surface and intracellular HLA-ABC expression. (B) HEK293T cells were transfected with plasmids encoding C-terminally Flag-tagged SARS-CoV ORF8a, SARS-CoV-2 ORF8 WT, or SARS-CoV-2 ORF8 C20A mutant. Forty-eight hours after transfection, cells were collected and analyzed for the cell surface HLA-ABC expression. Data are shown as the ratio of MFI in Flag+ cells to Flag- cells (n=3). Data are mean ± s.d. Data are representative of two to three independent experiments. **, $p < 0.01$; ***, $p < 0.001$

		Omicron subvariants				
		BA.1	BA.2.12.1	XAF	BA.4	BA.5
**Viral proteins**	E	**T9I**	**T9I**	**T9I**	**T9I**	**T9I**
	M	D3G<br>**Q19E**<br>**A63T**	**Q19E**<br>**A63T**	**Q19E**<br>**A63T**	**Q19E**<br>**A63T**	D3N<br>**Q19E**<br>**A63T**
	ORF7b				L11F	

Fig. S4. Mutations in SARS-CoV-2 viral proteins of Omicron subvariants
Non-synonymous mutations in SARS-CoV-2 Omicron subvariants found within viral proteins that we tested in Figure 3. Common mutations are shown in bold. GenBank Accession numbers for the reference sequences are as follows: BA.1: ON425981, BA.2.12.1: ON411581, XAF: OP031604, BA.4: ON773234, and BA.5: OP031606.

Yale SARS-CoV-2 Genome Surveillance Initiative members

Nicholas Chen, Mallery Breban, Anne M Hahn, Kien Pham, Tobias R Koch, Chrispin Chaguza, Irina Tikhonova, Christopher Castaldi, Shrikant Mane, Bony De Kumar, David Ferguson, Nicholas Kerantzas, David Peaper, Marie L Landry, Wade Schulz, Chantal BF Vogels, and Nathan D Grubaugh
