# Supplementary material for: Enhanced inhibition of MHC-I expression by SARS-CoV-2 Omicron subvariants
Source: Proc Natl Acad Sci U S A. 2023 Apr 10;120(16):e2221652120. doi: 10.1073/pnas.2221652120 (PMC10120007; doi:10.1073/pnas.2221652120)
Supplement: Supplementary file 2 — Dataset S01 (DOCX) [file pnas.2221652120.sd01.docx]

**Yale SARS-CoV-2 Genomic Surveillance Initiative members**

Nicholas Chen^1^, Mallery Breban^2^, Anne M Hahn^1^, Kien Pham^3^, Tobias R Koch^2^, Chrispin Chaguza^2^, Irina Tikhonova^2^, Christopher Castaldi^4^, Shrikant Mane^4^, Bony De Kumar^4^, David Ferguson^4^, Nicholas Kerantzas^5^, David Peaper^5^, Marie L Landry^5^, Wade Schulz^6^, Chantal BF Vogels^2^, and Nathan D Grubaugh^1,7^

1. Department of Epidemiology of Microbial Diseases, Yale School of Public Health, New Haven, CT, USA
2. Yale Institute for Global Health, Yale University, New Haven, CT, USA
3. Department of Pathology, Yale University School of Medicine, New Haven, CT, USA
4. Yale Center for Genome Analysis, Yale University, New Haven, CT, USA
5. Department of Laboratory Medicine, Yale New Haven Hospital, CT, USA
6. Center for Outcomes Research and Evaluation, Yale New Haven Hospital, CT, USA
7. Department of Ecology and Evolutionary Biology, Yale University, New Haven, CT, USA
